# Supplementary material for: Biodiversity and Safety Assessment of Half-Century Preserved Natural Starter Cultures for Pecorino Romano PDO Cheese
Source: Microorganisms. 2021 Jun 23;9(7):1363. doi: 10.3390/microorganisms9071363 (PMC8305336; doi:10.3390/microorganisms9071363)
Supplement: Supplementary file 1 [file microorganisms-09-01363-s001.zip › microorganisms-1220753-supplementary.pdf]

Supplementary Table S1 Primers used for specie-specific PCR, and the detection of antibiotic resistance genes, and pathogenic-related genes

Target	Sequence 5' – 3'	Annealing (°C)	Amplicon size (bp)	Reference
Enterococcus spp.	TACTGACAAACCATTCATGATG	59	112	[1]
	AACTTCGTCACCAACGCGAAC			
E. faecium	GAAAAAACAATAGAAGAATTAT	55	215	[2]
	TGCTTTTTTGAATTCTTCTTTA			
E. faecalis	ACTTATGTGACTAACTTAACC	55	360	[2]
	TAATGGTGAATCTTGGTTTGG			
E. durans	CCTACTGATATTAAGACAGCG	55	295	[2]
	TAATCCTAAGATAGGTGTTTG			
S. thermophilus	GCTTGTGTTCTGAGGGAAGC	58	577	[3]
	CTTTCTTCTGCACCGTATCCA			
L. delbrueckii subsp. *lactis*	TGCCAAGCTCTACTCCGTTT	56	217	[4]
	GTCAAGCGGCATAGTGTCAA			
L. reuteri	TGAATTGACGATGGATCACCAGTG	65	1000	[5]
	CGACGACCATGAACCACCTGT			
*tet*M	GAACTCGAACAAGAGGAAAGC	60	740	[6]
	ATGGAAGCCCAGAAAGGAT			
*tet*L	GTMGTTGCGCGCTATATTCC	55	696	[7]
	GTGAAMGRWAGCCCACCTAA			
*tet*S	GAAAGCTTACTATACAGTAGC	50	169	[6]
	AGGAGTATCTACAATATTTAC			

*tet*W	GAGAGCCTGCTATATGCCAGC	64	168	[6]
	GGGCGTATCCACAATGTTAAC			
*tet*K	TTATGGTGGTTGTAGCTAGAAA	55	348	[7]
	AAAGGGTTAGAAACTCTTGAAA			
*erm*A	TCAAAGCCTGTCGGAATTGG	58	440	[8]
	AAGCGGTAAACCCCTCTGAG			
*erm*B	CATTTAACGACGAAACTGGC	55	405	[8]
	GGAACATCTGTGGTATGGCG			
hyl_{efm}	GAGTAGAGGAATATCTTAGC	50	661	[9]
	AGGCTCCAATTCTGT			
esp	TTGCTAATGCTAGTCCACGACC	56	933	[10]
	GCGTCAACACTTGCATTGCCGAA			
*IS*16	CATGTTCCACGAACCAGAG	53	547	[11]
	TCAAAAAGTGGGCTTGGC			

N = A, C, G, and T; R = A and G; W = A and T; Y = C and T

Reference

1 Ke, D.; Picard, F.J.; Martineau, F.; Menard, C.; Roy, P.H.; Ouellette, M.; Bergeron, M.G. Development of a PCR assay for rapid detection of enterococci. *Journal of clinical microbiology* **1999**, *37*, 3497–3503.

2. Jackson, C.R.; Fedorka-Cray, P.J.; Barrett, J.B. Use of a genus- and species-specific multiplex PCR for identification of enterococci. *Journal of clinical microbiology* **2004**, *42*, 3558–3565, doi:10.1128/jcm.42.8.3558-3565.2004.

3. Lick, S.; Drescher, K.; Heller, K.J. Survival of Lactobacillus delbrueckii subsp. bulgaricus and Streptococcus thermophilusin the Terminal Ileum of Fistulated Göttingen Minipigs. *Applied and environmental microbiology* **2001**, *67*, 4137–4143, doi:10.1128/aem.67.9.4137-4143.2001.

4. Cremonesi, P.; Vanoni, L.; Morandi, S.; Silvetti, T.; Castiglioni, B.; Brasca, M. Development of a pentaplex PCR assay for the simultaneous detection of Streptococcus thermophilus, Lactobacillus delbrueckii subsp. bulgaricus, L. delbrueckii subsp. lactis, L. helveticus, L. fermentum in whey starter for Grana Padano cheese. *International journal of food microbiology* **2011**, *146*, 207–211, doi:10.1016/j.ijfoodmicro.2011.02.016.

5. Chagnaud, P.; Machinis, K.; Coutte, L.c.A.; Marecat, A.; Mercenier, A. Rapid PCR-based procedure to identify lactic acid bacteria: application to six common Lactobacillus species. *J Microbiol Methods* **2001**, *44*, 139–148, doi:10.1016/S0167-7012(00)00244-X.

6. Aminov, R.I.; Garrigues-Jeanjean, N.; Mackie, R.I. Molecular Ecology of Tetracycline Resistance: Development and Validation of Primers for Detection of Tetracycline Resistance Genes Encoding Ribosomal Protection Proteins. *Applied and environmental microbiology* **2001**, *67*, 22–32, doi:10.1128/aem.67.1.22-32.2001.
7. Gevers, D.; Danielsen, M.; Huys, G.; Swings, J. Molecular characterization of tet(M) genes in Lactobacillus isolates from different types of fermented dry sausage. *Applied and environmental microbiology* **2003**, *69*, 1270–1275, doi:10.1128/aem.69.2.1270-1275.2003.
8. Jensen, L.B.; Frimodt-Møller, N.; Aarestrup, F.M. Presence of erm gene classes in gram-positive bacteria of animal and human origin in Denmark. *FEMS microbiology letters* **1999**, *170*, 151–158, doi:10.1111/j.1574-6968.1999.tb13368.x.
9. Rice, L.B.; Carias, L.; Rudin, S.; Vael, C.; Goossens, H.; Konstabel, C.; Klare, I.; Nallapareddy, S.R.; Huang, W.; Murray, B.E. A potential virulence gene, hylEfm, predominates in Enterococcus faecium of clinical origin. *The Journal of infectious diseases* **2003**, *187*, 508–512, doi:10.1086/367711.
10. Eaton, T.J.; Gasson, M.J. Molecular screening of Enterococcus virulence determinants and potential for genetic exchange between food and medical isolates. *Applied and environmental microbiology* **2001**, *67*, 1628–1635, doi:10.1128/aem.67.4.1628-1635.2001.
11. Werner, G.; Fleige, C.; Geringer, U.; van Schaik, W.; Klare, I.; Witte, W. IS element IS16 as a molecular screening tool to identify hospital-associated strains of Enterococcus faecium. *BMC Infectious Diseases* **2011**, *11*, 80, doi:10.1186/1471-2334-11-80.
